# Integration of transcriptomics data into agent-based models of solid tumor metastasis

**DOI:** 10.1016/j.csbj.2023.02.014

**Published:** 2023-03-04

**Authors:** Jimmy Retzlaff, Xin Lai, Carola Berking, Julio Vera

**Affiliations:** a Laboratory of Systems Tumor Immunology, Department of Dermatology, Universitätsklinikum Erlangen and Friedrich-Alexander-Universität Erlangen-Nürnberg, Erlangen, Germany; b Deutsches Zentrum Immuntherapie, Erlangen, Germany; c Comprehensive Cancer Center Erlangen-EMN, Erlangen, Germany; d BioMediTech, Faculty of Medicine and Health Technology, Tampere University, Tampere, Finland

**Keywords:** Systems biology, Multi-scale modelling, Melanoma, Immunotherapy

## Abstract

Recent progress in our understanding of cancer mostly relies on the systematic profiling of patient samples with high-throughput techniques like transcriptomics. With this approach, one can find gene signatures and networks underlying cancer aggressiveness and therapy resistance. However, omics data alone cannot generate insights into the spatiotemporal aspects of tumor progression. Here, multi-level computational modeling is a promising approach that would benefit from protocols to integrate the data generated by the high-throughput profiling of patient samples. We present a computational workflow to integrate transcriptomics data from tumor patients into hybrid, multi-scale cancer models. In the method, we conduct transcriptomics analysis to select key differentially regulated pathways in therapy responders and non-responders and link them to agent-based model parameters. We then determine global and local sensitivity through systematic model simulations that assess the relevance of parameter variations in triggering therapy resistance. We illustrate the methodology with a *de novo* generated agent-based model accounting for the interplay between tumor and immune cells in a melanoma micrometastasis. The application of the workflow identifies three distinct scenarios of therapy resistance.

## 1 Introduction

In the last decade, the understanding of cancer pathogenesis and metastasis has progressed remarkably and improved our ability to diagnose, stratify and treat metastatic tumors. One hallmark of cancer is the ability of cancer cells to evade immune control [12]. The elucidation of this mechanism was vital to develop therapies such as immune checkpoint inhibitors (ICI), which have been successful with aggressive tumors like metastatic melanoma [19], [42]. Much of this progress in cancer research is due to the characterization of tissue samples from large cohorts of patients through genomics, transcriptomics, proteomics and/or epigenomics analysis. These techniques give access to quantitative data describing the activation and expression of (all) genes in cancer. This data provides the necessary foundation to investigate the genetic landscape of cancer progression [2] and to reconstruct and dissect the gene regulatory networks underlying cancer pathogenesis and therapy response [43], [7]. However, omics data alone cannot account for some levels of (de)regulation linked to spatiotemporal variations in the tumor’s molecular and cellular composition, as well as to the existence of nonlinear regulatory structures like feedback and feedforward loops [18].

In this context, mathematical modeling, in particular multi-level spatial computational models, is a viable method to investigate the dynamic behavior of the tumor microenvironment (TME) and to evaluate therapeutic strategies [25]. In these models, equations and other mathematical entities encode the cellular and molecular interactions between the cell types making up the tumor. One calibrates the values of the parameters in the equations utilizing biomedical knowledge and quantitative data from experiments. The parametrized models are then utilized to perform time-course simulations. In these simulations, one perturbs the amount of cancer cells in the tumor or the values of given model parameters, and simulates the dynamics of the tumor under these circumstances. These models can describe and predict the dynamics of cancer-deregulated intracellular gene circuits [16]. They can also integrate genes and gene circuits activity into tissue-scale models of cell-to-cell interactions [38], [41].

One type of multi-level spatial computational models are the agent-based models (ABM). In these models the tumor and immune cells are represented as discrete individuals that interact according to a pre-defined set of rules encoding their (patho)physiological behavior [29]. There are some challenges to devising these computational models in the context of cancer. First, there is a trade-off between detailed modeling of biological features with different scales occurring in cancer progression and keeping the model simple enough to allow interpretability and reasonable computing effort. Second, these models include a large amount of model parameters, and the calibration of their values requires diverse quantitative data. In the case of tissue modeling, many parameters can only be calibrated indirectly, as the experimental modalities to observe single cell behavior *in vivo* are missing. In line with this, quantitative characterization of 3D culture systems might enhance the capabilities to observe cellular interactions in tissues and cancer [10], [3].

When performing computational simulations representative of the pathophysiology of cancer, one has to carefully select which model parameter subset to analyze. One objective is to obtain computer simulations representative of the pathophysiological phenomenon of interest; a second, usually conflicting objective is to ensure that the simulations can be performed within a reasonable timeframe and computing effort. This parameter selection requires domain knowledge. Our idea is that transcriptomics data analysis can be employed to make this selection of relevant model parameters unbiased and systematic.

In this paper, we describe a methodology to decide the features of agent-based model simulations based on transcriptomics data analysis. In the methodology, one analyzes transcriptomics data to rank and select key gene sets underlying a condition of interest. We next link the gene sets to model parameters, and utilize this information to prioritize parameters for investigation of the model behavior. The influence of these prioritized parameters is investigated via global sensitivity analysis and large-scale, systematic model simulations. We exemplify the use of the method for a case study on melanoma metastasis and immunotherapy resistance. To this end, we built an agent-based model accounting for the interplay between tumor and immune cells in a micrometastasis.

## 2 Materials and methods

**Fig. 1:**
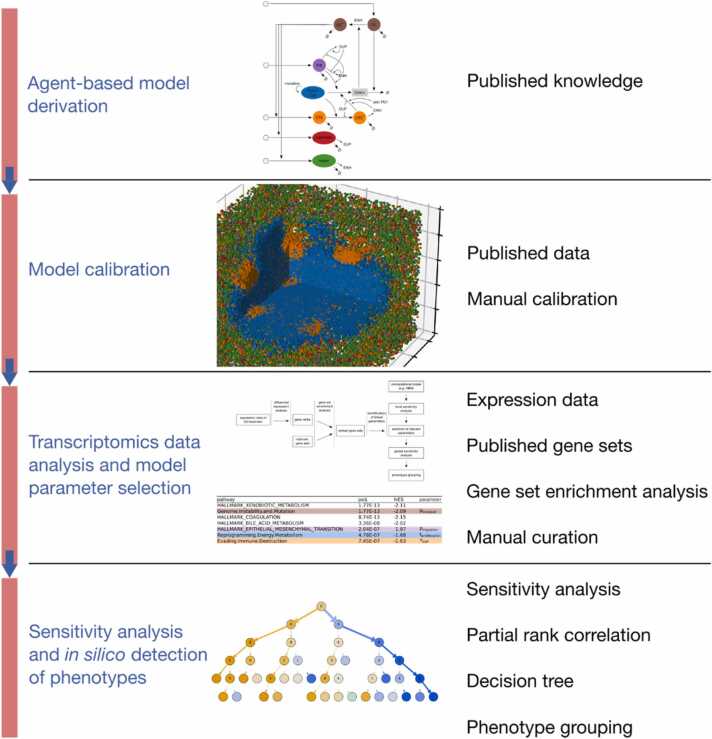
**Workflow of the study**. On the left are labels of the four overarching steps, on the right is a brief list of the central materials and methods used in each step. We first created the model and set a nominal parameter configuration. Then we linked expression data to model parameters to narrow a selection of parameters whose influence we analyzed in more depth.

**Workflow.**Fig. 1 depicts the workflow we followed in this study. It includes the following steps:

1. **Derivation of the agent-based model**: we collected knowledge on metastatic melanoma from publications, databases and published models to select the key cell types and biological processes involved in the interaction between cancer cells and the immune system. We encoded this information into the agents, rules and equations making up the model.
2. **Model calibration**: we combined manual curation of the literature to extract quantitative data with explorative model simulations to assign their nominal values to the model parameters.
3. **Transcriptomics data analysis and selection of key model parameters:** we collected and processed relevant transcriptomics datasets from tumor samples from ICI therapy responders and non-responders and performed differential expression analysis. Next, we performed gene set enrichment analysis to detect differentially regulated processes between responders and non-responders, and linked these processes to the computational model parameters. This information is employed to select model parameters of interest for sensitivity analysis.
4. **Sensitivity analysis and*****in silico*****detection of phenotypes:** we sampled the selected parameter space and performed systematic model simulations accounting for the reaction of the tumor micrometastasis to ICI therapy. To find sensitive parameter subspaces, we trained a decision tree. We quantified the parameter sensitivities utilizing partial rank correlation and feature importance derived from the decision tree. As target variable, we chose the cancer cell population at the simulation endpoint.

In the following, one can find a detailed explanation of the individual steps in the workflow.

### 2.1 Multi-level melanoma immunology model

The general modeling concept we used is to create a spatial agent-based model of the TME that interacts with a systemic compartment. This is a modeling approach that has been suggested in recent reviews [25], [29] and was followed in other multiscale models as well [11], [33]. We built our model based on knowledge of tumor immunology and signaling in cancer and melanoma [1], [24]. The model contains immune and tumor cells in the melanoma TME and their interactions, which can be either based on cell-cell contact or on intercellular communication through cytokines and other soluble factors.

**Fig. 2:**
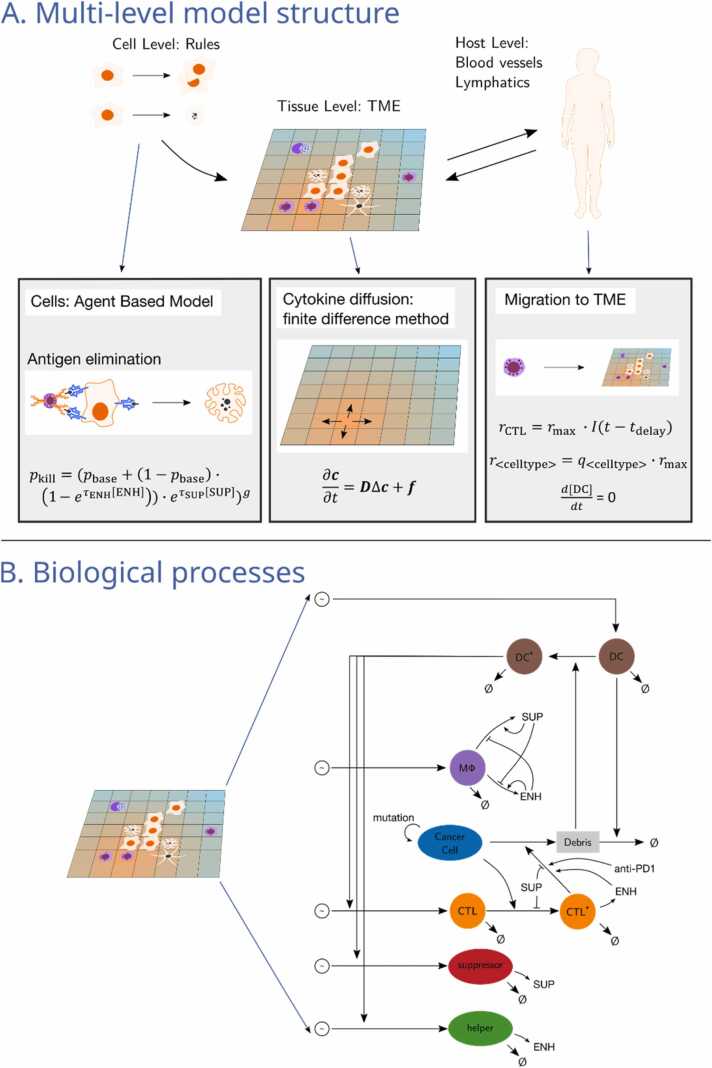
**Structure of the agent-based model. A: overview**. On the cellular level, an agent-based model is used, where cell physiology is described as cell type specific rules. This is coupled on the level of the tumor-microenvironment with the cytokine diffusion solver and the recruitment model of immune cells. **B: biological processes included in the model**. Cancer cell debris is detected by DCs which will attract CTLs, suppressors and helpers after a delay. CTLs that contact cancer cells switch to an activated state and can kill the cancer cell. The killing probability is influenced by cytokines and applied anti-PD1 therapy. Abbreviations: ENH: immunoenhancing cytokine, SUP: immunosuppressive cytokine, DC: dendritic cell, MΦ: macrophage, CTL: cytotoxic T lymphocyte.

The model accounts for parts of the innate and adaptive immunity to tumors including immunosurveillance without considering memory and long-term immunity. For the immunosurveillance we assumed that the tumor antigens are not yet detected by the adaptive immune system. A sketch of the model and in particular the considered cell interactions are shown in Fig. 2. Specifically, the immune cells accounted for are cytotoxic T lymphocytes (CTLs), T helper cells (Th), B cells, regulatory T cells (Tregs), dendritic cells (DCs), macrophages and myeloid-derived suppressor cells (MDSCs).

We assumed that the communication between immune cells is mediated by cytokines and chemokines. The cell behavior is modeled as logical rules. Further, our model includes helper cells and suppressor cells as abstract cell types that account for immune cells that primarily have a regulatory role, such as CD4 + T cells, B cells and MDSCs. These cells influence the immune response via secreting cytokines.

We labeled the involved cytokines based on whether they have a primarily immunoenhancing or immunosuppressive effect and modeled two abstract surrogate cytokines accordingly: immunoenhancing (ENH) and immunosuppressive cytokine (SUP). ENH accounts for cytokines that increase the effectiveness of cytotoxic mechanisms like IFN-γ, as well as for chemoattractants for cytotoxic cells such as CCL3, CCL4, CCL5, CXCL9 or CXCL10. Examples for molecular species that have immunosuppressive effects are IL-10, TGF-β and IDO. The surrogate cytokines keep the model simpler by assuming that the cytokines do not have pleiotropic effects, although it has been shown that some cytokines may trigger both immunosuppressive and immunoenhancing effects [6]. For instance, IFN-γ, a key regulator of the adaptive immune response, can trigger both the expression of major histocompatibility complex I (MHC-I) and of PD-L1. The former increases the recognition of cancer cells by T cells, while the latter inhibits the effector mechanism of T cells.

#### 2.1.1 Cytokine diffusion

Cytokines are modeled using a continuum model that tracks cytokine concentrations rather than discrete molecules. Their diffusion is described by Fick’s second law

$$\frac{\partial c}{\partial t}=D\Delta c+f$$

with the compounds’ concentrations ***c***, the diffusion constants ***D***, and a term ***f*** accounting for secretion and degradation. Cytokine diffusion is solved using a finite difference method with the Euler forward method.

#### 2.1.2 *In situ* cell populations

##### 2.1.2.1 Cancer cells

A cancer cell population of 125 cells is seeded at the lattice center at the beginning of a simulation. As the tumor cells migrate and divide, they will spread over the tissue in the course of a simulation.

Cell motility is implemented as a random walk, allowing a cell to move to neighboring lattice positions with a probability $p_{\mathrm{migration}}$. If the chosen position is already occupied by another cell, the cells either swap places or stay at their positions with equal probability. In general, it is assumed that cancer cells are less motile than immune cells.

Cancer cells can die with a probability $p_{\mathrm{death}}$, which leads to their removal from the lattice. Dead cancer cells will leave debris that can be collected by dendritic cells and facilitate an immune response.

Cancer cells are considered to have uncontrolled replication potential and will attempt to divide after a fixed length of time $t_{\mathrm{proliferation}}$ has passed, which accounts for cell growth and cycle duration. Dividing cells are temporarily immobile for the time step where the cell division occurs. Cell division can only take place if there is a vacant neighboring position that a daughter cell can occupy. This rule implicitly models cell contact inhibition, a trait that cancerous cells usually lose [12]. However, this assumption is in line with previous studies that use lattice-based models (e.g., [40]). It simplifies the modeling of cell mechanics, and evades calculations such as equilibria of forces between cells and the consequential possibility of cells pushing each other away.

Cancer cells may present one or multiple tumor-specific antigens depending on their mutations. We modeled only passenger mutations, meaning if a cancer cell mutates, it may start to present another antigen. This can induce an adaptive immune response specific to that antigen. Cancer cells mutate at each time step with a probability $p_{\mathrm{mutation}}$. The mutations that are modeled are non-driver mutations, as they only affect the antigen pool that a particular cancer cell presents, which influences its susceptibility towards clearance by CTLs: more mutations lead to a larger antigen pool and recognition by different CTL clones. The mutations are modeled as a finite allele model, with 32 possible mutations. This allows to track the individual mutations, but has the disadvantage that it is not realistic compared to the near-infinite mutations possible in a real human genome, potentially leading to artifacts, e.g., through the possibility of reverse mutations.

##### 2.1.2.2 Cytotoxic T lymphocytes

Cytotoxic T lymphocytes patrol the tumor site and are able to induce apoptosis in cancer cells upon contact. A cell is considered to be in contact with another if it is present in its Moore neighborhood (i.e., adjacent cell including diagonal adjacency). A CTL recognizes an antigen that is specific to its receptor and kills cancer cells presenting the antigen. It probes its neighborhood for a recognizable cancer cell in random order. The randomness is introduced to avoid a direction bias that might lead to simulation artifacts. If the CTL recognizes a cancer cell, it kills it with a probability modeled as

$$p_{\mathrm{kill}}={\left(\left(p_{\mathrm{base}}+\left(1-p_{\mathrm{base}}\right)\left(1-e^{-\tau_{\mathrm{ENH}}\left[\mathrm{ENH}\right]}\right)\right)e^{-\tau_{\mathrm{SUP}}[\mathrm{SUP}]}\right)}^{g}$$

that depends on a base killing probability $p_{\mathrm{base}}$, the local concentrations of SUP [SUP] and ENH [ENH], respective rate constants $\tau_{\mathrm{SUP}}$ and $\tau_{\mathrm{ENH}}$, and the influence of anti PD1 checkpoint inhibitor therapy g. The probability is modeled in such a way that killing of tumor cells by CTLs becomes more effective in the presence of ENH and less effective in the presence of SUP. Anti PD1 therapy is modeled as a power law influence [37] and is set to 1 (no therapy) and can be toggled to 0.1 during a simulation (application of therapy). At this abstraction level of the model, it is indifferent whether the drug targets PD1 which may be expressed by CTLs or its ligand PDL1 which may be expressed by the cancer cells, as it only influences the killing mechanism on contact. The killing has a duration $t_{\mathrm{kill}}$, in which the CTL becomes immobile and unable to kill other neighboring cancer cells.

CTLs undergo apoptosis after a fixed lifespan $t_{\mathrm{life},\mathrm{CTL}}$ . Note that CTL expiration accounts for different cell fates including exhaustion, apoptosis or leaving the TME. We do not model CTL proliferation in the TME, although it has been reported in cases of combination therapy [36]. Unlike cancer cells, a CTL follows its migration rule at every time step unless it is in an immobile state. Therefore, its motility depends only on the cell density in its vicinity. It is capable of performing both random walk and chemotactic migration, following the ENH gradient. By default, CTLs perform random walk and they change to chemotactic migration if the concentration of surrounding ENH cytokines exceeds a threshold. We modeled this threshold to prevent the CTLs from being sensitive to very low ENH concentrations. The CTLs moving in the chemotactic mode are in an activated state. The activated CTLs secrete ENH cytokines with a fixed rate $r_{\mathrm{ENH}}$. ENH cytokines increase their cytotoxic capabilities and attract other CTLs to their vicinity, allowing fast finding and clearance of cancer cell colonies.

##### 2.1.2.3 Dendritic cells

DCs are antigen-presenting cells to immune effector cells such as T cells. In the model, DCs function as probes for tumor cells, and they move inside the lattice at each simulation step if a vacant position in the vicinity is available. We did not consider apoptosis of DCs in the TME, as we assume that DCs are either tissue-resident or filtrated through the TME during their lifetime. A DC will collect all cancer cell debris it encounters. It then starts to present the antigens it processed. Furthermore, it becomes activated and leaves the tumor site. Once a DC has left the tumor site, it increases a signal that leads to a delayed recruitment of CTLs that are specific to the antigens it now presents. This way we implicitly modeled the homing of DCs to the tumor-draining lymph node. We assume homeostasis of the DC population, and for every DC that leaves the tumor microenvironment, a new DC will be recruited.

##### 2.1.2.4 Helper and suppressor cells

Helper cells account for CD4 + helper T cells as well as tumor infiltrating B cells. They constantly secrete ENH cytokines ($r_{\mathrm{ENH}}$). Suppressor cells primarily account for regulatory T cells (Treg) and myeloid derived suppressor cells (MDSCs). Analog to helper cells, they constantly secrete SUP cytokines. Both helper and suppressor cells perform a random walk during their lifetime, which is fixed to $t_{\mathrm{life},\mathrm{helper}}$ and $t_{\mathrm{life},\mathrm{suppressor}}$, respectively.

##### 2.1.2.5 Macrophages

Macrophages have cytotoxic capabilities, and can secrete both ENH and SUP cytokines. Similar to CTLs, their cytotoxicity is influenced by cytokines. Their cytokine secretion rates depend on the ratio of the concentrations of local SUP and ENH cytokines:

$$r_{\mathrm{SUP}}=\frac{\left[\mathrm{SUP}\right]}{\left[\mathrm{SUP}\right]+\left[\mathrm{ENH}\right]}\cdot r_{\mathrm{cytokine}}$$

and

$$r_{\mathrm{ENH}}=\frac{\left[\mathrm{ENH}\right]}{\left[\mathrm{SUP}\right]+\left[\mathrm{ENH}\right]}\cdot r_{\mathrm{cytokine}}$$

The equations result in positive feedback loops, making the secretion rates of SUP and ENH by macrophages positively correlate with their own concentrations. The feedback loops imitate an environment-dependent phenotype plasticity that resembles M1 and M2 phenotype activation described in the literature [15].

#### 2.1.3 Cell recruitment

Newly recruited cells appear on a free position at the border of the TME lattice based on the assumption that recruited cells enter from nearby blood vessels. The cell types recruited to the TME are CTLs, DCs, macrophages, helper and suppressor cells.

The recruitment of CTLs is preceded by DC-induced clonal expansion and differentiation in the lymphatic tissues, which introduces a delayed response. Therefore, CTLs are recruited to the TME with an antigen specific rate $r_{\mathrm{CTL}}$ that depends on delayed tumor detection of DCs.

The delay is modeled as a queue with a fixed size $t_{\mathrm{delay}}$ for each antigen. At each simulation step, the oldest value will be dequeued and leads to recruitment of CTLs, while a new value will be enqueued and initialized to zero. Every DC presenting the respective antigen that leaves the tumor site at the simulation step will add a number to the new value in the queue, leading to immune cell recruitment in the future. Helper cells are recruited alongside CTLs with a fixed ratio of 1:1, which approximates reported data (Hernberg 1996).

Recruitment of suppressor cells and macrophages depend on CTL recruitment with fixed ratios $q_{<\mathrm{celltype}>}$, that is

$$r_{\mathrm{macrophage}}=q_{\mathrm{macrophage}}\cdot r_{\mathrm{CTL}}.$$

### 2.2 Model environment, simulation and parameterization

The lattice is modeled with cubic cells with a side length of 10μm, with 100×100×100 cells, representing a volume of $1\;{\mathrm{mm}}^{3}$. We set Δt=10min for the duration of a simulation step, which is taken from a similar model by Gong et al. [11]. The fastest action a cell undertakes and that is affected by the step size is cell movement. The time step and cell length correspond with a maximum cell speed of $\sqrt{3}\;\mathrm{\mu m}/\min$. The maximum cell speed is therefore about 10 times slower than the speed of neutrophils performing chemotaxis in a microfluidic device and about 5 times faster than H69 small cell lung cancer cells [26]. We assume this depicts cell motion with an adequate speed assuming that leukocytes move more slowly in tissue than in a microfluidic device. Simulations start with a small homogeneous population of 125 cancer cells at the center of the lattice and random uniformly distributed populations of DCs and macrophages. We run simulations for a period of 100 days or 8401 steps, respectively. This period is chosen on the assumption that a successful immune response will clear the metastasis within 50 days, as is indicated for adaptive immune responses [1]. We doubled the simulation time to investigate the model progression of small residual cancer cell populations that many simulations showed at day 50. Further, a simulation will abort earlier if the cancer cell population grows larger than 800,000. This abortion condition is chosen to limit the computational effort of the simulations. We consider it justified as 80% of the lattice spaces will be occupied by cancer cells, effectively simulating a tumor expansion beyond the model space.

We used published experimental data to calibrate as many parameters as possible, whose annotation and nominal values are listed in supplementary Table S1. To determine the maximum recruitment rates, we use ratios of cell types that are described in literature, leaving but one recruitment rate uncharacterized. With this modeling choice we achieve that the simulated immune infiltrate resembles an infiltrate found in experiments over the course of a simulation.

Running a single simulation took about 1 h and 20 min on our hardware (4 Intel Xeon E5–4660, 256 GB RAM), requiring about 10 MB RAM. For the sensitivity analyses, we ran up to 64 simulations in parallel. We implemented our model in c+ +17, using HDF5 and json data formats for I/O. To account for the stochasticity in the model, we repeated each simulation multiple times with different seeds for the random number generator. To decide on a number of replicas to make for each simulation, we ran simulations with the nominal parameter configuration both with and without application of ICI therapy and compared the 95% confidence intervals of the expected cancer cell populations after 3 and 100 replications. Following this procedure, we found that using a low number of replications is acceptable for our analysis (Supplementary Fig. S1). For the local sensitivity we repeated each simulation 10 times, and for the global sensitivity analysis 3 times.

### 2.3 Linking differential regulation to model parameters

**Fig. 3:**
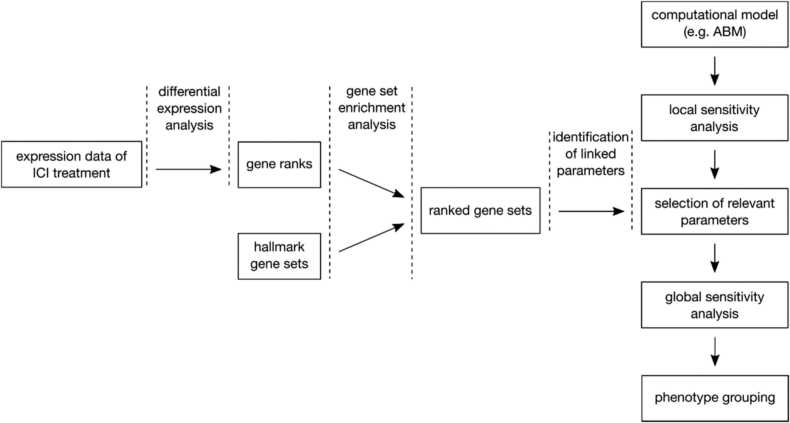
**Method of linking expression data to model parameters**. Besides performing a local sensitivity analysis to preselect a set of parameters for global analysis, we propose to enrich expression data of different conditions to link them to parameters of potential biological relevance.

For computationally expensive model simulations, global sensitivity analysis is only feasible for a small subset of model parameters. Here we propose to base the selection on parameters linked to biologically relevant gene sets using a workflow as depicted in Fig. 3. We performed a gene set enrichment analysis to identify and characterize a subset of them that is related to response to anti-PD1 treatment. First, we downloaded and processed the transcriptome data of pre-treatment melanomas undergoing anti-PD1 checkpoint inhibition therapy (GSE78220). Second, we identified differentially expressed genes between responders and non-responders. Third, we performed a gene set enrichment analysis using the differentially expressed genes and identified gene sets in which the genes are involved. We assumed that the identified enriched gene sets are crucial for the pathogenesis and progression of melanoma, and therefore we manually annotated them with corresponding model parameters.

For the gene set enrichment analysis, we used the R package fgsea [35] that tested the enrichment of the identified differentially expressed genes using the MSigDB hallmark gene set collection [21] and cancer hallmark genes (CHG) [44]. The fgsea algorithm searches for gene sets where highly ranked genes are enriched. It is given a ranked list of genes and a list of gene sets. We calculated gene ranks based on differential expression as

$$s_{i}=\mathrm{sign}\left(\mathrm{logF}C_{i}\right)\cdot \left(-\log_{10}p_{i}\right)$$

with the binary logarithmic fold change $\mathrm{logF}C_{i}$ and the p-Value $p_{i}$. To explore the enrichment, we examined the Benjamini-Hochberg adjusted p-value and listed the significantly regulated gene set. These were manually annotated with model parameters they relate to, excluding those that could not be related (e.g., because of their generality or association to processes that are not modeled).

Next, we selected parameters for global sensitivity analysis based on the gene set enrichment and the local sensitivity analysis. In our case, we could afford to run a global sensitivity analysis for 5 parameters, while 6 parameters were identified with the gene set enrichment. To exclude one parameter from this selection, we performed a local sensitivity analysis (i.e., one at a time perturbation) and excluded the least influential parameter.

### 2.4 Sensitivity analysis and decision tree-based phenotype grouping

We selected five model parameters of interest for sensitivity analysis, which we set up in a quasi-Monte Carlo fashion where we sample the selected parameter space using the Sobol’ sampling sequence implementation of chaospy [9]. As boundaries for the parameter space we set (0, 2) times the nominal value. Assuming that about five simulations per parameter are needed to sufficiently cover the parameter space, we sample $5^{5}=3125$ parameter sets. As the model includes stochastic processes such as killing or moving probabilities, we repeat each simulation 3 times, leading to a total number of 9375 simulations for the sensitivity analysis.

To analyze the sensitivity of our parameter selection, we trained a decision tree as a meta-model, intending to find sensitive parameter subspaces on the simulation outcome [13], [31], [32]. A similar approach has been followed in an earlier work [34]. To quantify the parameter sensitivities, we used partial rank correlation and feature importance derived from the decision tree [23]. As target variable we chose the cancer cell population at the end of the simulation, labeling the simulation results either as emerging metastasis (>700,000), complete remission (0) or else residual disease. This gives an indication of how good the immune response is in eliminating the emerging metastasis. There are some limitations though, as we can generally not assume that a simulation will reach a steady-state by its end.

Decision trees have the advantage that they can reproduce nonlinear and non-monotonous behavior, which is useful as we cannot assume a linear model behavior a priori. We use the scikit-learn implementation, which also calculates normalized parameter importance on the regression splits [30]. As an optimization criterion we chose the Gini impurity. The simulations were randomly split into a training and test data set (80:20) and 5-fold cross-validation was carried out on the training set yielding a mean accuracy of 0.9 and a standard deviation of 0.004. To avoid overfitting and to keep the decision tree easily human-interpretable we constrain it to a depth of 5 and a minimum split size of 3.3%.

## 3 Results

Our research aim was to utilize systematic simulations of an agent-based model to detect *in silico* phenotypes linked to the resistance of metastatic melanoma to ICI therapy. To this end, we derived and calibrated an agent-based model describing the interplay between cancer and immune cells in a tumor micrometastasis. The selection of model parameters perturbed in the simulations is critical as a trade-off between biomedical meaningfulness and computing effort. To tackle this, we developed a computational methodology to make an unbiased selection of model parameters based on transcriptomics data analysis.

### 3.1 Model derivation and selection of the nominal model configuration

We developed an agent-based model of the immune reaction to melanoma that considers the core cellular mechanisms influenced by the cytokine milieu. The TME is set up to simulate a newly seeded micrometastasis, where a cancer cell colony a) grows, b) is detected by immunosurveillance and c) is challenged by both innate and CTL-mediated adaptive immune responses (see Material and Methods for details).

**Fig. 4:**
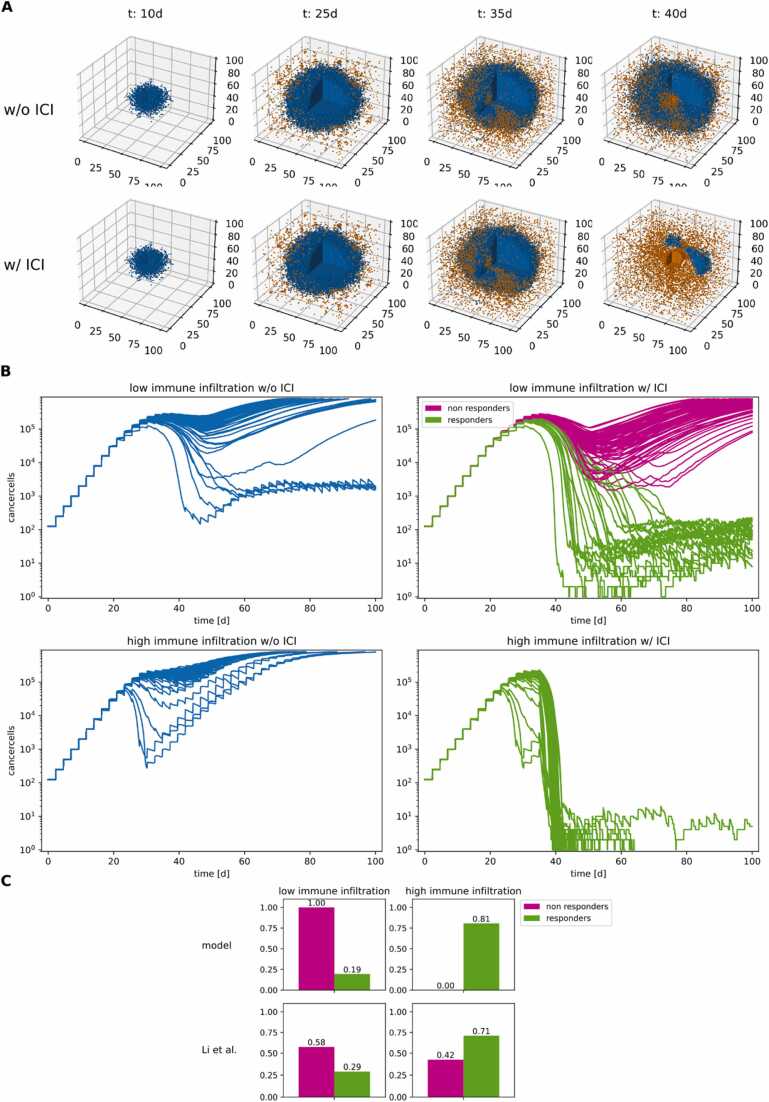
**Simulations of the nominal parameter configuration with and without ICI therapy**. **A:** comparison of the cell lattices over the course of a simulation. Only cancer cells (blue) and CTLs (orange) are shown for clarity. **B:** cancer cell populations of 100 simulations per condition with randomly perturbed model parameters (+/- 25%). High immune infiltration marks the nominal configuration, low immune infiltration simulations have a 3-fold reduced immune cell recruitment rate. The simulations with ICI are designated “responders” or “non-responders” depending on their final cancer cell population. **C:** qualitative comparison of the conditions with fractions of anti-PD1 responders and non-responders with high and low CD8 infiltrates respectively (For interpretation of the references to colour in this figure legend, the reader is referred to the web version of this article).

We calibrated most model parameters to data estimates from the literature (Supplementary Table S1). For three parameters, we did not find data. To address this issue, we explored their parameter space to find a sensitive parameter set, which we fixed as nominal values (Supplementary Fig. S2). In Fig. 4 we show simulations of the nominal parameter configuration and a configuration with reduced recruitment of immune cells with and without ICI treatment. The design of the simulations is motivated by findings indicating that immune cell infiltration, in particular CTL infiltration, correlates with ICI treatment outcome [17], [20], [28], [8]. In the simulations, we randomly perturbed the model parameter values within the range of + /- 25% of their nominal values to account for patient diversity. For the nominal parameter configuration, simulations without ICI lead to emerging metastases in almost every case. However, simulations with low CTL infiltration and application of ICI lead to remission in 76/100 and to residual disease in 24/100 simulations. Finally, simulations with ICI and the nominal (high) CTL infiltration led to complete removal of cancer cells in 99/100 simulated cases and residual disease in one case. Taken together, the selection of parameter values for the nominal model configuration renders results that qualitatively match clinical evidence of patient response with high and low CTL infiltration ([20], see Fig. 4 C).

### 3.2 Transcriptomics data-driven selection of therapy-response related gene sets and their connection to model parameters

**Fig. 5:**
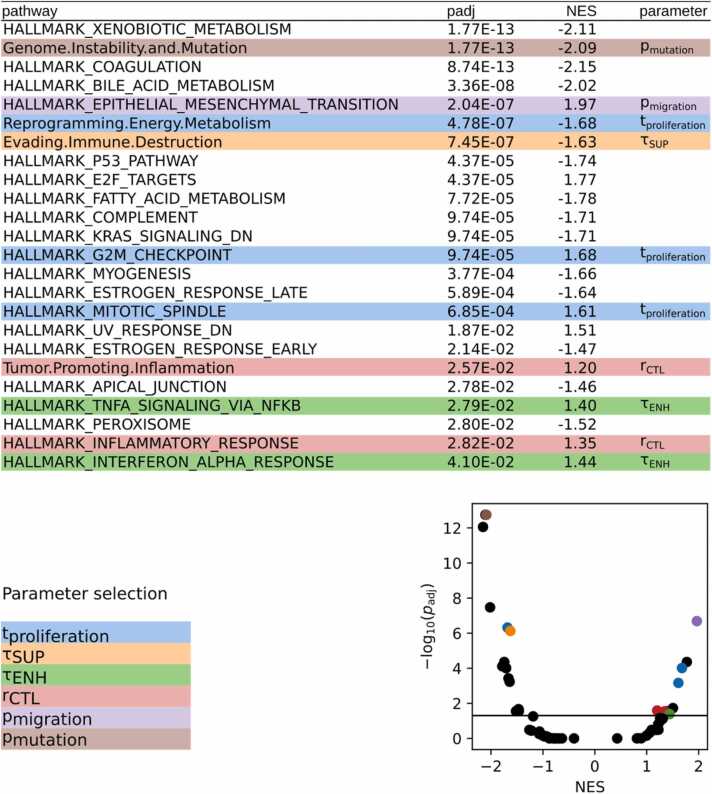
**Selection of model parameters that are related to differential regulation in therapy responders vs. non-responders**. **Top:** significantly differentially regulated gene sets in melanoma sample of different ICI treatment response that could be linked to model parameters. See full list in supplementary Table 2. **Bottom left:** list of the identified parameters connected to the selected gene sets. **Bottom right:** volcano plot of the adjusted p value against the normalized enrichment score. Gene sets accounting for parameters are marked with corresponding colors.

To find molecular differences between responders and non-responders to ICI therapy, we downloaded and processed the transcriptome data of pre-treatment melanomas from patients undergoing anti-PD1 checkpoint inhibition therapy (GSE78220). Gene set enrichment analysis using differential gene expression (anti-PD1 responders versus non-responders) resulted in 24 significantly differentially regulated gene sets (Benjamini-Hochberg adjusted p-value<= 0.05). Fig. 5 illustrates how we used this data to identify relevant model parameters, which we selected for the computationally expensive global analysis. For the parameter selection we considered the significantly differentially regulated gene sets. We annotated and mapped these genes sets to the corresponding model parameters. We excluded 14 gene sets that could not be linked to any model parameter because they are generic, disease-specific, or not directly related to any modeled mechanism.

We linked the gene set “*genome instability and mutation*” to the mutation probability ($p_{\mathrm{mutation}}$). We linked “*epithelial-mesenchymal transition*” to cancer cell motility ($p_{\mathrm{migration}}$). We connected three gene sets to the cell cycle time ($t_{\mathrm{proliferation}}$), and one to the influence of suppressive cytokines ($\tau_{\mathrm{SUP}}$.). We linked two gene sets to the CTL recruitment rate ($r_{\mathrm{CTL}}$). Finally, we connected two other gene sets to the influence of enhancing cytokines ($\tau_{\mathrm{ENH}}$).

### 3.3 Parameter sensitivity analysis indicates multiple mechanisms of therapy resistance

**Fig. 6:**
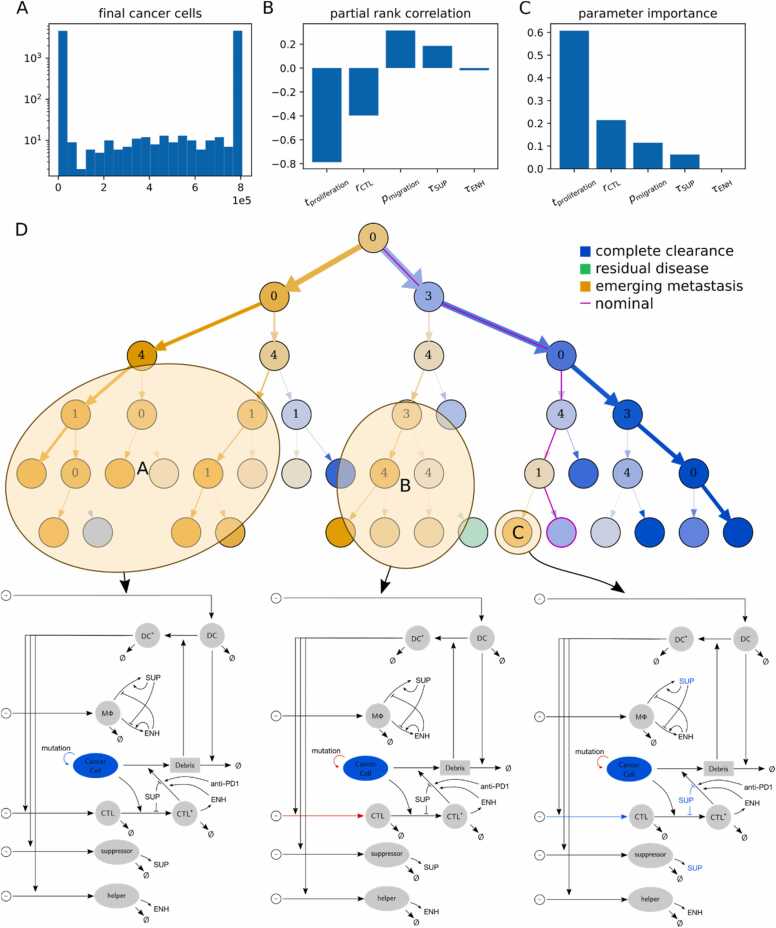
**Decision tree-based sensitivity analysis. A:** distribution of final cancer cell populations. **B:** partial rank correlation coefficients**. C:** parameter importance of the decision tree. **D:** decision tree for the final cancer cell population. Each node predicts a simulation outcome for a region of the sampled parameter space. At a branch parent node, the parameter space is split along a threshold for a split parameter. The numbers in the parents refer to split parameters: 0: cell cycle time of cancer cells $t_{\mathrm{proliferation}}$, 1: influence of immunosuppressive cytokines τ, 3: CTL recruitment rate $r_{\mathrm{CTL}}$, 4: migration probability of cancer cells $p_{\mathrm{migration}}$. The color saturation indicates node impurity. Model drafts are shown for highlighted ICI resistant parameter subspaces with color indicating model components influenced by parameter deviations from the nominal configuration (blue: lower values; red: higher values). (For interpretation of the references to colour in this figure legend, the reader is referred to the web version of this article.)

To account for limited computational power encountered in large-scale ABM, we constrained the global sensitivity analysis to five parameters. We excluded the enriched parameter $p_{\mathrm{mutation}}$ because it is the least influential of the selected parameters in the local sensitivity analysis performed (cf. supplementary Fig. S3). Global sensitivity analysis showed that a large proportion of simulations ended either with an emerging metastasis or complete remission (Fig. 6 A). Based on these results, we assigned each simulation to one of the categories “*emerging metastasis*”, “*residual disease*” and “*complete remission*”. Fig. 6 B/C shows the influence of the selected parameters. We quantified this influence utilizing the partial rank correlation coefficients (prccs) and the parameter importance of the decision tree (Fig. 6. B/C). The results with both metrics suggest that cancer cell cycle duration and motility are more influential than the CTL recruitment rate and the cytokine-associated parameters. The signs of the prccs can be interpreted intuitively: simulations with long cell cycle times, high CTL recruitment rates and high influence of enhancing cytokines ENH lead to better removal of cancer cells. However, in simulations with higher cancer cell motility and higher influence of suppressive cytokines SUP the removal of cancer cells is compromised.

The decision tree indicates that the simulated ICI therapy is effective in multiple conditions of the TME. However, it also reveals different mechanisms of therapy resistance (Fig. 6. D, Supplementary Fig. 4). In case of the ICI therapy-resistant decision tree regions, we include in Fig. 6 a model sketch with the model processes whose parameters are modified in color. In the decision tree-based classification of the model simulations, the tumor tends to be resistant to ICI therapy in the following cases: i) aggressive tumors with short replication time and at least moderate motility, ii) tumors with longer replication time but high motility and smaller CTL recruitment, and iii) tumors with higher CTL recruitment and longer cell replication time, but high influence of suppressive cytokines SUP and high cancer cell motility. These findings suggest that the difficulty in predicting ICI therapy response [27] may be due to a spectrum of existing counter-balancing mechanisms that influence ICI therapy effectiveness.

## 4 Discussion

Our aim was to develop an approach to integrate transcriptomic data into computational models of cell-to-cell interactions in cancer. There is abundant scientific literature exploring the use of unsupervised and supervised machine-learning models and omics data to classify cancer patient samples [[39], [5]]. However, few studies have explored the integration of these data with tissue-level mechanistic computational models. This combination allows for computer model-supported interpretation of patient data. To this end, we implemented a hybrid, agent-based model describing the interplay between cancer and immune cells in melanoma micrometastasis. To build and characterize the model, we used knowledge of melanoma immunology and publicly available quantitative data describing the behavior of the melanoma cells and different immune cells infiltrating the TME.

There are similar cancer models proposed in the literature. Wang et al. [40] developed an agent-based melanoma model accounting for cytokine-mediated angiogenesis. Hatzikirou et al. [14] modeled tumor invasion with a lattice gas cellular automaton. Gong et al. [11] modeled the tumor immune response to PD-1/PD-L1 inhibition. They identified tumor mutational burden and antigen strength as critical factors that influence the recruitment of immune cells. They simulate therapy with checkpoint inhibition by changing a model parameter (probability of T cell suppression) at a set time point during a simulation, the same approach we use to model therapy. Compared to the model proposed in this work, we do not model CTL proliferation at the TME (compare with section 2.1.2). Instead, our model considered a greater extent of cell types, including DCs, helper and suppressor cells, and variability in tumor antigens. While this increases the complexity of the model, it hypothetically allows for a more detailed projection of the differential regulation data into the model. The model’s limitations arise from abstraction and simplification, but they are also due to incomplete knowledge on the cellular mechanisms. For instance, the finite allele mutation model does not replicate the significance of mutational burden on the prognosis of ICI [27].

Here we combined gene set enrichment analysis of cancer immunotherapy response data and global sensitivity analysis of systematic model simulations to focus the investigation on selected model parameters. The motivation for this is that in large multi-level computational models, one cannot analyze the parameter space exhaustively due to limitations in computational power. In our case, the systematic exploration of the entire parameter space would require about $5^{28}$ simulations. However, the proposed method reduced the effort to 9375 simulations and about 175 h of computation. Previous approaches utilized hypothesis-driven parameter selection for deciding model simulations, but our method performs parameter selection in an unbiased, omics-data-driven fashion.

Based on our analysis, we suggest a causal relationship between given differentially regulated gene sets and cellular phenotypes associated with selected cell types in the TME. In the case of our model, the investigation of the designated parameters identified three different mechanisms of ICI treatment resistance. Distinctively activated cellular processes in these resistance mechanisms are: a) cancer cell motility, previously suggested as a mechanism of resistance [7]; b) CTL infiltration, known to play a role in discriminating the immune "hot" and "cold" tumors [22]; and c) suppressing immune signals, which can be mediated by TGFβ [45], IDO [4] and others.

A limitation of the method is that the linkage between parameters and gene sets remains a manual curation step and depends on the expert knowledge of the modelers. Further, the enrichment analysis could, in principle, detect gene sets not linked to any parameter in the current instance of the model. In this case, one can utilize our approach to perform a data-driven, systematic model expansion. For example, our analysis found three gene sets related to cellular metabolism differentially regulated between responders and non-responders. One can utilize this information to expand the model with equations accounting for cancer and immune cell metabolism and the diffusion of nutrients in the TME.

Our data-parameter linkage approach is based solely on gene set enrichment analysis and remains a qualitative step, yielding a categorical classification of parameters. A quantitative differentiation of parameters would enable us to deduce parameter perturbations directly from the data. In this regard, one can expand the method to generate a quantitative link of transcriptomic data to parameters. To this end, one would calculate the magnitude of the differential regulation and map it as a perturbation level to the respective parameters relative to their nominal calibration. However, this requires annotation of all parameters with their associated gene sets. This task is currently intensive in manual work but could be alleviated by automated methods such as text mining.

We think that the combination of enrichment analysis of transcriptomics data and global sensitivity analysis can be applied generally to agent-based or ODE models reflecting cell-to-cell and tissue interactions in cancer and other pathologies (cf. Fig. 3). To this end, it is necessary to have a significant amount of annotated transcriptomics data reflecting the investigated conditions or progression of the disease. When selecting the number of parameters to be explored, one has to consider a trade-off between sufficient sampling of the chosen sub-parameter space and keeping the required computational load in control.

## Ethics

This article does not contain any studies involving human or animal participants.

## Funding

This work has been supported by the German Ministry of Education and Research (BMBF) through the initiatives e:Med-MelAutim on cancer and autoimmunity [01ZX1905A to XL and JV] and KI-VesD on computation modelling and artificial intelligence guided cancer diagnostics [031L0244A to JV]. We also received funding for our computer model-based research in melanoma from the Manfred Roth-Stiftung, the Trunk-Stiftung and the Matthias-Lackas-Stiftung.

## Author statement

The idea was developed by JV. The funding was acquired by XL and JV. The ABM model derivation and the simulation study were performed by JR under the supervision of JV and XL. All the authors drafted, reviewed and edited the document.

## Conflicts of interest

We have no conflicts of interest to disclose. The funders had no role in the design, analysis, write-up or decision to submit for publication.

## Data Availability

The code of the model generated for this work is publicly accessible on https://doi.org/10.5281/zenodo.6277598.
