# Supplementary material for: Integration of transcriptomics data into agent-based models of solid tumor metastasis
Source: Comput Struct Biotechnol J. 2023 Mar 4;21:1930–41. doi: 10.1016/j.csbj.2023.02.014 (PMC10024179; doi:10.1016/j.csbj.2023.02.014)
Supplement: Supplementary file 1 — Supplementary material [file mmc1.pdf]

Supplementary Material for: Integration of transcriptomics data into agent-based models of solid tumor metastasis

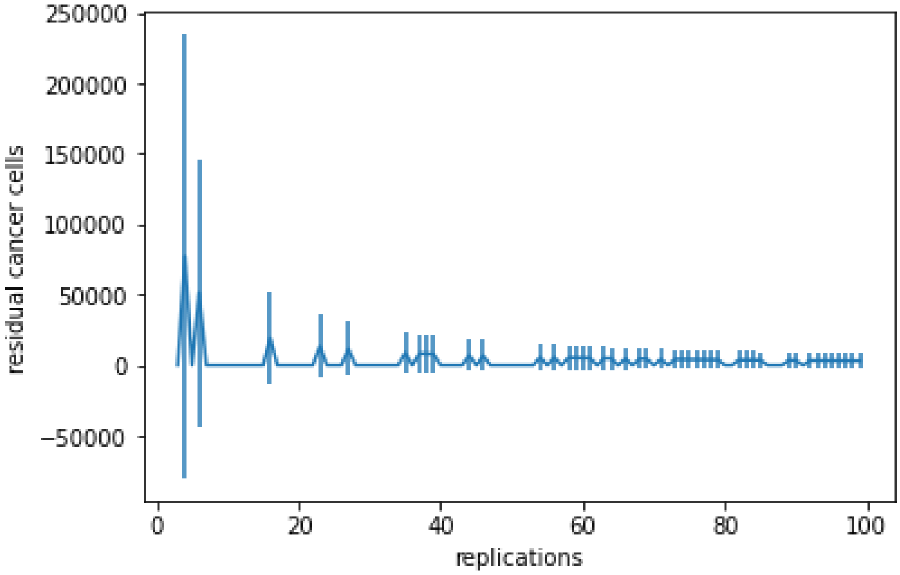

Supplementary figure S1: Estimation of mean cancer cell populations at the end of simulations. The errorbars denote the 95% confidence interval.

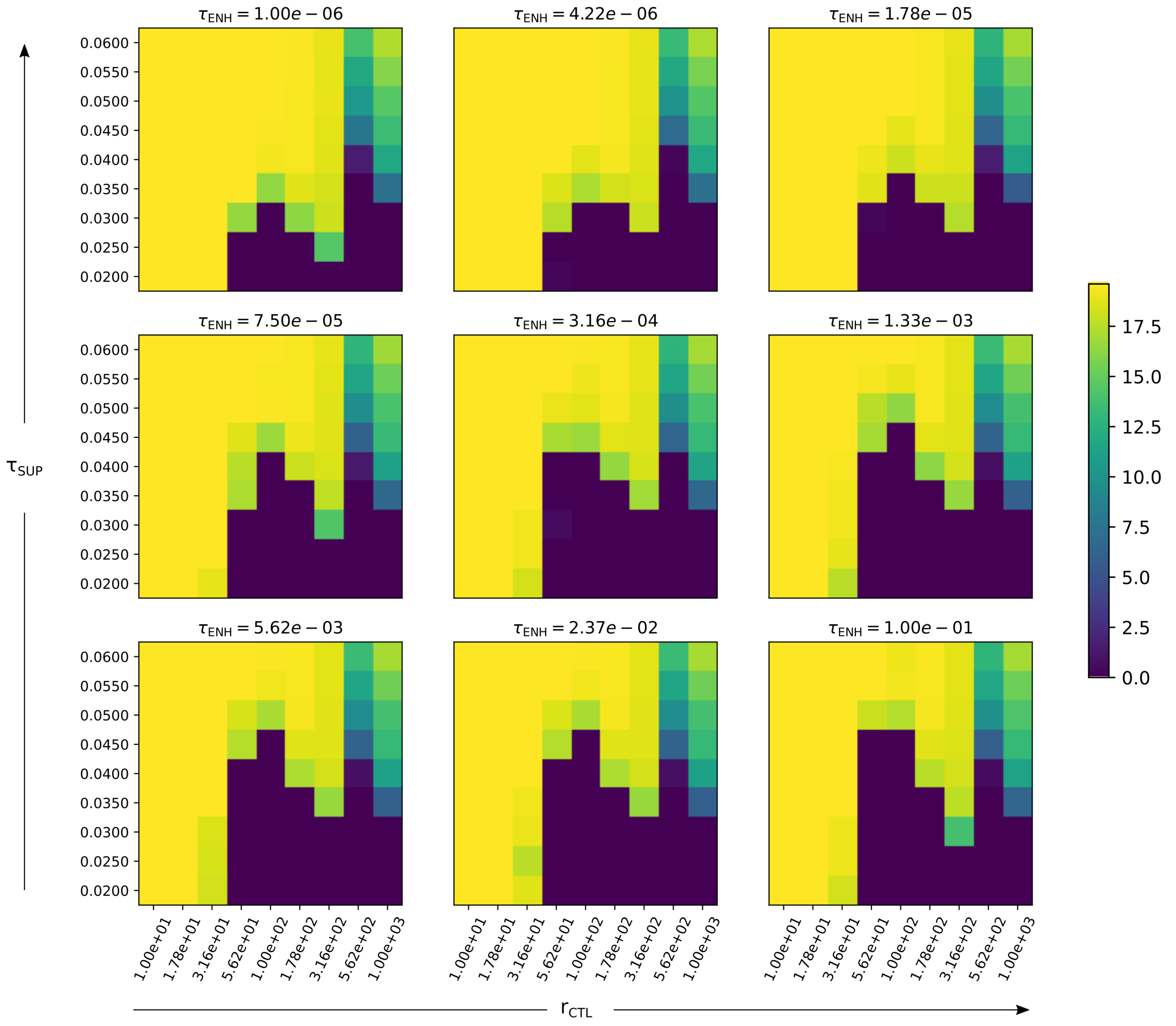

Supplementary figure S2: Parameter space of the uncalibrated parameters. The binary logarithm of the final cancer cell population is color coded.

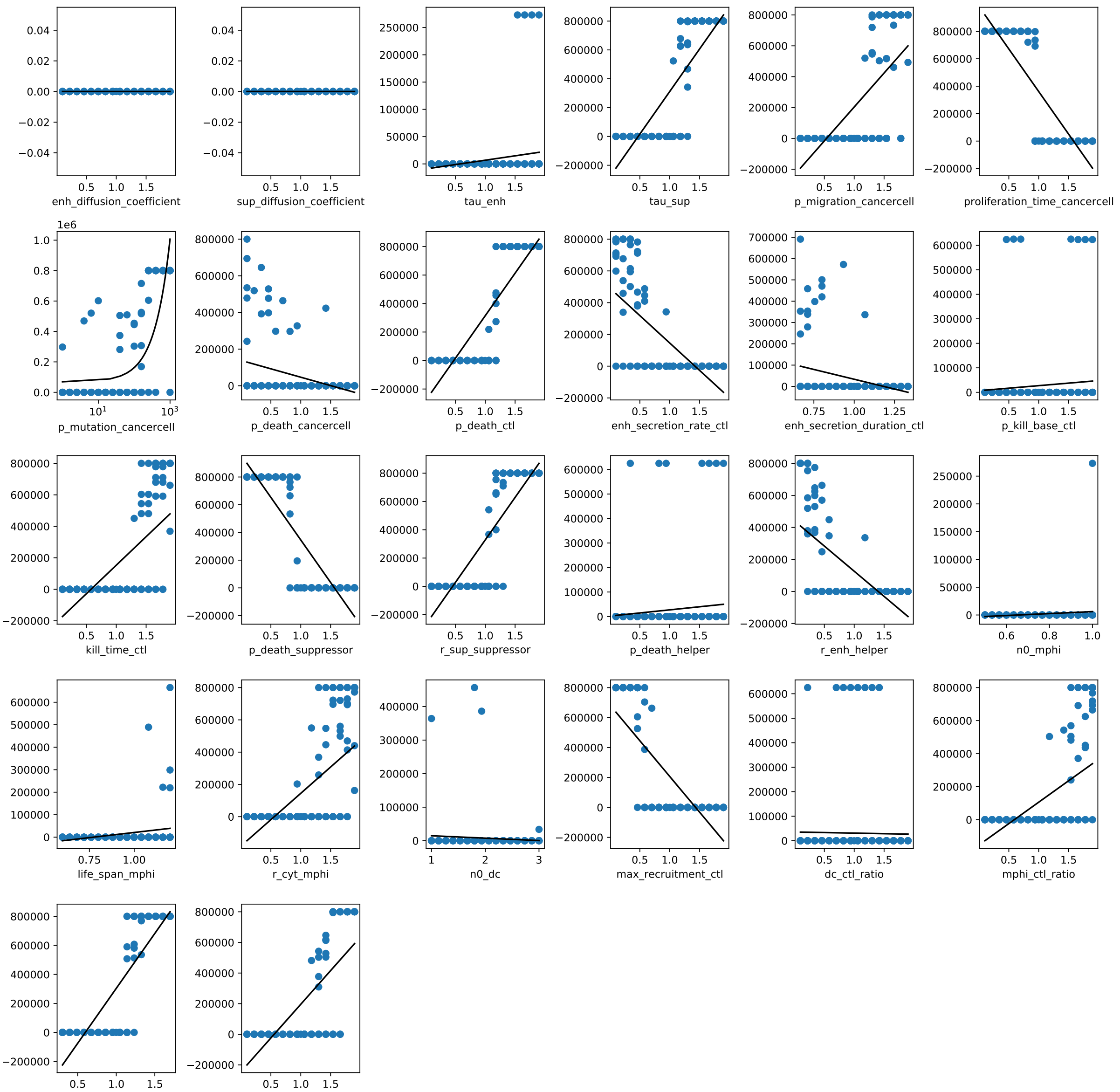

Supplementary figure S3: Local sensitivity analysis of the model parameters. The black lines denote linear regressions.

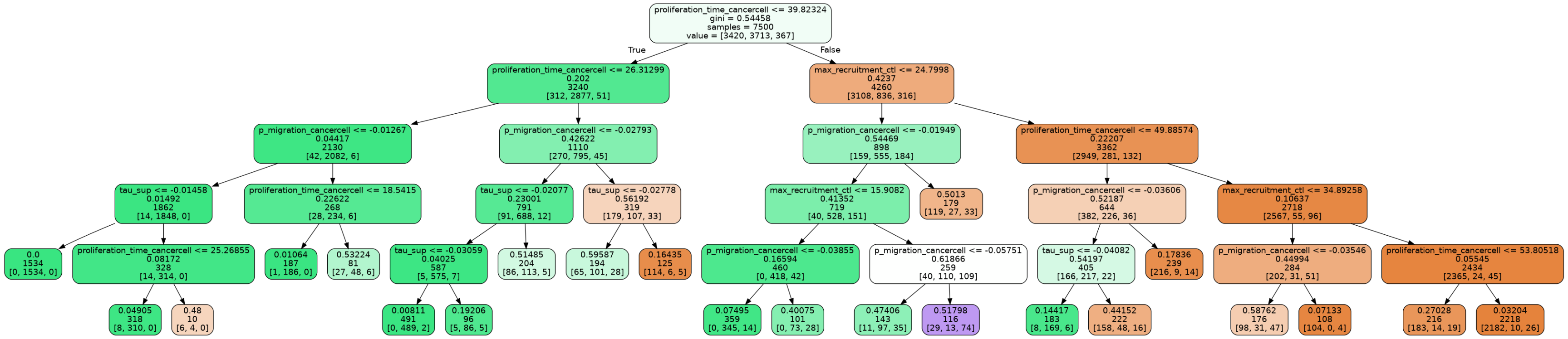

Supplementary figure S4: Decision tree with detailed information on the splits. Values for τ_{SUP} and $p_{\text{migration}}$ are negated. The first line of a node contains the split parameter and threshold, the second its gini impurity, the third the number of simulations that are accounted by the node, and the fourth line the number of simulations in the different classes [remission, metastasis, residual disease].

Supplementary table 1: Summary of the model parameters. *Parameter estimation from data in the reference.

Parameter	Description	Nominal	Interval	Reference
Cell framework				
l	Cell side length	$10\,\mu\mathrm{m}$		[9]
	Lattice size	$100 \times 100 \times 100 (1\,\mathrm{mm}^3)$		[9]
Δt	Time step	$10\,\mathrm{min}$		[9]
Cytokines				
D_{ENH}	Diffusion coefficient for ENH	$36\,000\,\mu\mathrm{m}^2\,\mathrm{h}^{-1}$		[3]
D_{SUP}	Diffusion coefficient for SUP	$36\,000\,\mu\mathrm{m}^2\,\mathrm{h}^{-1}$		[3]
τ_{ENH}	Influence of ENH on the kill probability	0.01		estimated
τ_{SUP}	Influence of SUP on the kill probability	0.04		estimated
Cancer cells				
$p_{\mathrm{migration}}$	Moving probability of cancer cells	0.05		[14]*
$t_{\mathrm{proliferation}}$	Cell cycle time of cancer cells	$46\,\mathrm{h}$		[14], [2]
p_{mutation}	Mutation probability of cancer cells per gene per cell division	10^{-6}	$[10^{-6}, 10^{-3}]$	[14], [6]
p_{death}	Dying probability of cancer cells	0.0002		[13]
CTLs				
$p_{\mathrm{death,CTL}}$	Dying probability of CTLs at the TME	0.0028		[5]
r_{ENH}	Secretion rate of ENH by CTLs	$6000\,\mathrm{h}^{-1}$		[11]
t_{ENH}	Secretion duration of ENH by CTLs	$3\,\mathrm{h}$	[2, 4]	[4]
$p_{\mathrm{kill,base}}$	Base kill probability of CTLs	1/3		[18]
t_{kill}	CTL inactivation time after killing a cancer cell	$50\,\mathrm{min}$		[10]
Suppressors				
$p_{\mathrm{death,supressor}}$	Dying probability of suppressor cells at the TME	0.0006		[17]
r_{SUP}	Secretion rate of SUP by suppressor cells	$6000\,\mathrm{h}^{-1}$		[11]
Helper				
$p_{\mathrm{death,helper}}$	Dying probability of helper cells at the TME	0.0006		[17]
r_{ENH}	Secretion rate of ENH by helper cells	$6000\,\mathrm{h}^{-1}$		[11]
Macrophages				
$n_{0,\mathrm{macrophage}}$	Initial macrophage population size	500	[250, 500]	[8]
$t_{\mathrm{life,macrophage}}$	Life span of macrophages at the TME	$120\,\mathrm{h}$	[72, 144]	[15]
r_{cytokine}	Secretion rate of cytokines by macrophages	$6000\,\mathrm{h}^{-1}$		[11]

Continued on next page

Parameter	Description	Nominal	Interval	Reference
DCs				
$n_{0,\mathrm{DC}}$	Initial DC population size	1000	[1000, 3000]	[16]
Recruitment				
r_{CTL}	Recruitment rate of CTLs to the TME	$100\,\mathrm{h}^{-1}$	[10, 1000]	
$q_{\mathrm{macrophage}}$	Macrophage / CTL ratio	$0.37 \cdot r_{\mathrm{CTL}}$		[7]
q_{Tcells}	Treg / CTL ratio	0.076	$[0.023, 0.129]$	[12]
t_{delay}	Delay between DC antigen report and CTL recruitment	$168\,\mathrm{h}$		[1]

Supplementary table 2: Enrichment of Hallmark gene sets in anti-PD1 responders vs. non-responders (GSE78220). Significant results (padj < 0.05) are annotated with model parameters.

pathway-enrichment-GSE78220

	pathway	pval	padj	log2err	ES	NES	size	parameter
55	HALLMARK_XENOBIOTIC_METABOLISM	5.8996765204063E-15	1.76990295612189E-13	0.996986217533181	-0.787889543790827	-2.10692052317677	197	none
5	Genome.Instability.and.Mutation	3.93852744784999E-15	1.76990295612189E-13	0.996986217533181	-0.779905886562157	-2.08910623317467	216	p_mutation
15	HALLMARK_COAGULATION	4.36961862665697E-14	8.73923725331394E-13	0.965327754226083	-0.829290945274524	-2.15389992515777	137	none
13	HALLMARK_BILE_ACID_METABOLISM	2.24270774194028E-09	3.36406161291043E-08	0.774939030136436	-0.794792264617605	-2.0177784415499	112	none
19	HALLMARK_EPITHELIAL_MESENCHYMAL_TRANSITION	1.70132189173136E-08	2.04158627007763E-07	0.73376198835648	0.620203208083549	1.96579778162797	195	p_migration
57	Reprogramming.Energy.Metabolism	4.78266835384818E-08	4.78266835384818E-07	0.719512826338911	-0.601719955041389	-1.68253646374854	443	cell cycle time
4	Evading.Immune.Destruction	8.69381860777817E-08	7.45184452095272E-07	0.704975715167238	-0.574318497572625	-1.62650744463243	586	tau_sup
42	HALLMARK_P53_PATHWAY	6.29050725153679E-06	4.37479724540042E-05	0.610526878385931	-0.653777208541138	-1.74433538366838	192	none
18	HALLMARK_E2F_TARGETS	6.56219586810063E-06	4.37479724540042E-05	0.610526878385931	0.559353076117229	1.77292703721374	195	none
22	HALLMARK_FATTY_ACID_METABOLISM	1.28601896478468E-05	7.71611378870808E-05	0.593325476396405	-0.679397216532026	-1.78271648070086	156	none
16	HALLMARK_COMPLEMENT	2.11001801319693E-05	9.73854467629354E-05	0.575610261071129	-0.638595850319474	-1.70949392149987	199	none
33	HALLMARK_KRAS_SIGNALING_DN	2.0700072720978E-05	9.73854467629354E-05	0.575610261071129	-0.640927434127702	-1.70884086590784	194	none
23	HALLMARK_G2M_CHECKPOINT	2.05118221669488E-05	9.73854467629354E-05	0.575610261071129	0.531879302736931	1.67877896765981	190	cell cycle time
39	HALLMARK_MYOGENESIS	8.78796516956346E-05	0.000376627078696	0.538434096309916	-0.621660595858179	-1.66328982753649	198	none
21	HALLMARK_ESTROGEN_RESPONSE_LATE	0.000147232276257	0.000588929105029	0.518848077743792	-0.614405222180608	-1.64057837042737	196	none
35	HALLMARK_MITOTIC_SPINDLE	0.000182592790313	0.000684722963672	0.518848077743792	0.506421461343078	1.60978158781805	197	cell cycle time
52	HALLMARK_UV_RESPONSE_DN	0.005291171117345	0.01867472159063	0.407017918923954	0.495074612186953	1.50549005953459	138	none
20	HALLMARK_ESTROGEN_RESPONSE_EARLY	0.006424522630287	0.021415075434291	0.407017918923954	-0.549728582086951	-1.46912837493279	195	none
60	Tumor.Promoting.Inflammation	0.008139296271105	0.025703040856121	0.380730400722792	0.337717233573597	1.20100779686102	620	max_recruitment_ctl
10	HALLMARK_APICAL_JUNCTION	0.009269061373386	0.027807184120157	0.380730400722792	-0.548989604367774	-1.46387356556655	193	none
50	HALLMARK_TNFA_SIGNALING_VIA_NFKB	0.009766593253799	0.027904552153711	0.380730400722792	0.438763070423865	1.39554540033859	198	tau_enh
44	HALLMARK_PEROXISOME	0.010252803780436	0.027962192128463	0.380730400722792	-0.607925333561322	-1.51927544064341	104	none
30	HALLMARK_INFLAMMATORY_RESPONSE	0.010822749461056	0.028233259463624	0.380730400722792	0.4236442901761	1.34665457561982	197	max_recruitment_ctl
31	HALLMARK_INTERFERON_ALPHA_RESPONSE	0.01640640778502	0.041016019462551	0.352487857583619	0.500859791657303	1.44045244582158	95	tau_enh
59	Sustaining.Proliferative.Signaling	0.023046092184369	0.055310621242485	0.30068212900741	-0.413550259272206	-1.18823753735596	1257	
36	HALLMARK_MTORC1_SIGNALING	0.030283875915986	0.069885867498429	0.352487857583619	0.396431971956246	1.25653186065591	195	
32	HALLMARK_INTERFERON_GAMMA_RESPONSE	0.03365784430014	0.074795209555866	0.321775918075361	0.411453729173949	1.30868433992441	198	
37	HALLMARK_MYC_TARGETS_V1	0.044196982670606	0.094707820008441	0.321775918075361	0.400010705753871	1.27090497254369	194	
48	HALLMARK_SPERMATOGENESIS	0.074257425742574	0.153636053260498	0.376039306993639	0.406025689572192	1.22187751992126	132	
49	HALLMARK_TGF_BETA_SIGNALING	0.165562913907285	0.32258064516129	0.199915231309662	0.449105480647152	1.18279092541582	54	
54	HALLMARK_WNT_BETA_CATENIN_SIGNALING	0.166666666666667	0.32258064516129	0.186432558434075	0.479444305518297	1.22632883794447	42	
25	HALLMARK_HEDGEHOG_SIGNALING	0.174311926605505	0.326797385620915	0.127505315300183	-0.591177997236322	-1.25965534902625	34	
2	Enabling.Replicative.Immortality	0.185185185185185	0.326797385620915	0.323470511553299	0.327673386973156	1.09431907785968	299	
29	HALLMARK_IL6_JAK_STAT3_SIGNALING	0.184873949579832	0.326797385620915	0.213927855492356	0.414482299518257	1.1711138650371	87	
9	HALLMARK_ANGIOGENESIS	0.211480362537764	0.362537764350453	0.11331290842208	-0.566312286803746	-1.22069517036301	36	
58	Resisting.Cell.Death	0.244979919678715	0.408299866131191	0.080419996876154	-0.370715240108163	-1.06462850675795	1145	
28	HALLMARK_IL2_STAT5_SIGNALING	0.30379746835443	0.492644543277455	0.204294756516886	0.331780476331377	1.05161230361103	195	
3	Evading.Growth.Suppressors	0.339487179487179	0.536032388663968	0.06494076846129	-0.370347975765586	-1.05436860900179	661	
14	HALLMARK_CHOLESTEROL_HOMEOSTASIS	0.358208955223881	0.551090700344432	0.076279718080927	-0.44899504657103	-1.07172981411457	73	
11	HALLMARK_APICAL_SURFACE	0.453592814371257	0.680389221556886	0.069283646739255	-0.457859969692331	-1.00720146450371	43	
27	HALLMARK_HYPOXIA	0.488649940262843	0.698071343232634	0.054907370329096	-0.370898757573757	-0.988211935132508	191	
40	HALLMARK_NOTCH_SIGNALING	0.485875706214689	0.698071343232634	0.10027910675413	0.415527621832798	0.995274280728132	32	
34	HALLMARK_KRAS_SIGNALING_UP	0.541120381406436	0.755051694985725	0.050092287440211	-0.36101694421271	-0.962646937482929	193	
56	Inducing.Angiogenesis	0.576759061833689	0.776447105788423	0.042077210988939	-0.344817717588742	-0.968023769925494	485	
43	HALLMARK_PANCREAS_BETA_CELLS	0.582335329341317	0.776447105788423	0.057246113520288	-0.425975218704307	-0.926972169304473	39	
26	HALLMARK_HEME_METABOLISM	0.605952380952381	0.790372670807453	0.044864511316856	-0.347290558938431	-0.926601910338933	192	
7	HALLMARK_ALLOGRAFT_REJECTION	0.720379146919431	0.919632953514168	0.036685042761344	-0.326948822430929	-0.873758083235641	195	
45	HALLMARK_PI3K_AKT_MTOR_SIGNALING	0.784105960264901	0.972145615002758	0.03792714671081	-0.330190176990654	-0.825183289705759	104	
38	HALLMARK_MYC_TARGETS_V2	0.793918918918919	0.972145615002758	0.08243440915271	0.306867361044419	0.828571720318353	58	
12	HALLMARK_APOPTOSIS	0.821621621621622	0.985945945945946	0.107972360317345	0.289628063139072	0.894820462675071	159	
1	Activating.Invasion.and.Metastasis	0.887210473313192	1	0.016838053046391	-0.305028153685303	-0.875143862528585	1102	
6	HALLMARK_ADIPOGENESIS	0.896428571428571	1	0.02618415159462	-0.294301874693068	-0.785223416785341	192	
53	HALLMARK_UV_RESPONSE_UP	0.906288532675709	1	0.027448008180102	-0.29234860415355	-0.767653307450578	154	
24	HALLMARK_GLYCOLYSIS	0.938315539739027	1	0.023508484501329	-0.283691784971784	-0.758629237720138	197	
41	HALLMARK_OXIDATIVE_PHOSPHORYLATION	0.984468339307049	1	0.021145636251359	-0.267145829551459	-0.711018394854852	184	
46	HALLMARK_PROTEIN_SECRETION	0.969855832241153	1	0.02709674231833	-0.277099010616261	-0.686106707802994	94	
8	HALLMARK_ANDROGEN_RESPONSE	0.993394980184941	1	0.026254339649827	-0.255947489501375	-0.633525127498473	96	
17	HALLMARK_DNA_REPAIR	1	1	0.022726926034007	-0.153287435758237	-0.400531437285294	147	
47	HALLMARK_REACTIVE_OXYGEN_SPECIES_PATHWAY	1	1	0.066896627650067	0.167076110181607	0.431288020210968	47	
51	HALLMARK_UNFOLDED_PROTEIN_RESPONSE	0.905172413793103	1	0.08862611332851	0.276124013534668	0.811302214045898	107	

References

[1] Abul K Abbas, Andrew HH Lichtman, and Shiv Pillai. *Cellular and Molecular Immunology.* eighth. Elsevier Health Sciences, 2014. ISBN: 978-0-323-22275-4.

[2] Renato Baserga. *The biology of cell reproduction.* Harvard University Press, 1985.

[3] Dorothea Busse et al. "Competing feedback loops shape IL-2 signaling between helper and regulatory T lymphocytes in cellular microenvironments". In: *Proceedings of the National Academy of Sciences* 107.7 (2010), pp. 3058–3063.

[4] Samuel CC Chiang et al. "Comparison of primary human cytotoxic T-cell and natural killer cell responses reveal similar molecular requirements for lytic granule exocytosis but differences in cytokine production". In: *Blood, The Journal of the American Society of Hematology* 121.8 (2013), pp. 1345–1356.

[5] Rob J De Boer, Dirk Homann, and Alan S Perelson. "Different dynamics of CD4+ and CD8+ T cell responses during and after acute lymphocytic choriomeningitis virus infection". In: *The Journal of Immunology* 171.8 (2003), pp. 3928–3935.

[6] Peter Duesberg, Reinhard Stindl, and Rüediger Hehlmann. "Explaining the high mutation rates of cancer cells to drug and multidrug resistance by chromosome reassortments that are catalyzed by aneuploidy". In: *Proceedings of the National Academy of Sciences* 97.26 (2000), pp. 14295–14300.

[7] Gulsun Erdag et al. "Immunotype and immunohistologic characteristics of tumor-infiltrating immune cells are associated with clinical outcome in metastatic melanoma". In: *Cancer research* 72.5 (2012), pp. 1070–1080.

[8] Reinhild Feuerstein et al. "Resident macrophages acquire innate immune memory in staphylococcal skin infection". In: *Elife* 9 (2020), e55602.

[9] Chang Gong et al. "A computational multiscale agent-based model for simulating spatio-temporal tumour immune response to PD1 and PDL1 inhibition". In: *Journal of The Royal Society Interface* 14.134 (2017), p. 20170320.

[10] Stephan Halle et al. "In vivo killing capacity of cytotoxic T cells is limited and involves dynamic interactions and T cell cooperativity". In: *Immunity* 44.2 (2016), pp. 233–245.

[11] Qing Han et al. "Multidimensional analysis of the frequencies and rates of cytokine secretion from single cells by quantitative microengraving". In: *Lab on a chip* 10.11 (2010), pp. 1391–1400.

[12] Joannes FM Jacobs et al. "Regulatory T cells in melanoma: the final hurdle towards effective immunotherapy?" In: *The lancet oncology* 13.1 (2012), e32–e42.

[13] Xiao-Hong Ma et al. "Measurements of tumor cell autophagy predict invasiveness, resistance to chemotherapy, and survival in melanoma". In: *Clinical cancer research* 17.10 (2011), pp. 3478–3489.

[14] R Milo et al. "BioNumbers–the database of key numbers in molecular and cell biology". In: *Nucleic Acids Res* 38.Database issue (Jan. 2010). BNID 106815, 112247, pp. 750–753. DOI: 10.1093/nar/gkp889. URL: https://www.ncbi.nlm.nih.gov/pmc/articles/PMC2808940/.

[15] Debbie C Strachan et al. "CSF1R inhibition delays cervical and mammary tumor growth in murine models by attenuating the turnover of tumor-associated macrophages and enhancing infiltration by CD8+ T cells". In: *Oncoimmunology* 2.12 (2013), e26968.

[16] Mascha J Toebak et al. "Dendritic cells: biology of the skin". In: *Contact dermatitis* 60.1 (2009), pp. 2–20.

[17] Diana L Wallace et al. "Direct measurement of T cell subset kinetics in vivo in elderly men and women". In: *The Journal of Immunology* 173.3 (2004), pp. 1787–1794.

[18] Bettina Weigelin et al. "Cytotoxic T cells are able to efficiently eliminate cancer cells by additive cytotoxicity". In: *Nature communications* 12.1 (2021), pp. 1–12.
